# Research Progress of Laccase in Edible and Medicinal Fungi

**DOI:** 10.3390/jof12050350

**Published:** 2026-05-08

**Authors:** Yanshu Zhao, Xiaojia Zhang, Yuxin Jiang, Shuyuan Song, Chuang Han, Xiaodong Dai

**Affiliations:** Institute of Microbiology, Heilongjiang Academy of Sciences/National Collection of Edible Fungi (Heilongjiang), Harbin 150010, China; zhaoyanshu0213@163.com (Y.Z.);

**Keywords:** edible and medicinal fungi, laccase gene, gene cloning, expression regulation, biocatalysis

## Abstract

This paper systematically reviews the research progress on the physiological functions, gene cloning, classification basis, and expression regulation mechanisms of laccase in edible and medicinal fungi. Laccase is a copper-containing polyphenol oxidase widely distributed in these fungi, characterized by broad-spectrum substrate catalytic activity and redox properties. It plays a central role in lignin degradation, pigment synthesis, and environmental pollutant treatment. Regarding gene cloning, researchers have successfully isolated and identified laccase genes from multiple species using techniques such as transcriptome sequencing, RACE amplification, and gene knockout. Expression regulation studies have revealed that laccase genes exhibit stage-specific expression patterns during mycelial growth, fruiting body development, and lignin degradation. In recent years, breakthroughs in genomics, transcriptomics, and gene editing technologies have greatly advanced research into the cloning, classification, and regulatory mechanisms of laccase genes. This article systematically reviews the diversity, clonal classification, and regulatory mechanisms of these genes, aiming to provide a reference for further research and industrial development of laccase in edible and medicinal fungi.

## 1. Introduction

Fungal laccases are a class of blue multicopper oxidoreductases that catalyze single-electron transfer from both phenolic and nonphenolic compounds [1]. Unlike lignin peroxidase and manganese peroxidase, fungal laccase does not require H_2_O_2_ for substrate oxidation; instead, it utilizes O_2_ as the sole co-substrate, offering advantages such as simplicity, high efficiency, green chemistry, energy savings, and environmental friendliness [2]. Furthermore, fungal laccases can function in synergy with small-molecule redox mediators. After the mediator is rapidly oxidized by laccase, gaining one or more electrons, the oxidized mediator diffuses out of the laccase catalytic pocket and oxidizes substrate molecules, thereby promoting the oxidative cleavage of recalcitrant substrates such as macromolecular lignin [3].

Lignin, the most abundant renewable aromatic biopolymer on Earth, is rapidly emerging as a key driver of next-generation sustainable technologies. Lignin-derived carbon materials offer low-cost alternatives to essential raw materials for batteries. The pharmaceutical industry is increasingly harnessing lignin’s unique properties to develop bio-based, clean-label products. Meanwhile, lignin-based adsorbents provide efficient and biodegradable solutions for removing persistent environmental pollutants. Supported by global sustainability roadmaps such as the European Green Deal and China’s 14th Five-Year Plan, and fueled by unprecedented investment and cross-sector collaboration, lignin is evolving from an industrial residue into a strategic asset. Breakthroughs in lignin upgrading, intelligent formulation, and application-driven design are overcoming long-standing barriers related to scale, performance, and standardization. Lignin is no longer merely a promising biopolymer—it is a catalytic force accelerating the global transition toward a circular, climate-resilient, and green industrial transformation. The future of sustainable innovation is being built with lignin [4]. The primary component of food and agricultural waste is lignin—the most abundant biopolymer in nature—which features a complex structure, strong recalcitrance, and low degradability [5]. Therefore, efficiently decomposing lignin in agricultural waste has become a key scientific challenge. Current pretreatment methods for lignin include physical, chemical, and biological approaches. Among these, biological methods have attracted considerable attention due to their green, economical, and environmentally friendly characteristics [6]. Extracellular laccase secreted by white rot fungi has been shown to depolymerize lignin into cellulose, hemicellulose, and other carbohydrates, thereby significantly improving the biofermentability of lignin-derived oxidation products while reducing energy consumption and waste streams [7]. When assisted by small-molecule mediators, fungal laccase can achieve lignin decomposition rates as high as 80–90% [8]. Clearly, using laccase-producing fungi for liquid or solid fermentation can not only induce laccase biosynthesis and production but also facilitate the conversion of lignin into bioenergy and industrially valuable chemicals. To date, the most extensive research on lignin oxidative decomposition by extracellular laccase has focused on white rot fungi such as *Agaricus bisporus* and *Pleurotus ostreatus* [9]. Laccase is generally considered a secondary metabolite of fungi [1]. In recent years, breakthroughs in genomics, transcriptomics, and gene editing technologies have driven significant progress in understanding the cloning, classification, and expression regulation mechanisms of laccase genes. In this review, we summarize the diversity, clonal classification, and regulatory mechanisms of laccases, aiming to provide a reference for further research and industrial development of laccases derived from edible and medicinal fungi.

## 2. Physiological Functions of Laccase

### 2.1. Structural Features

**Figure 1:**
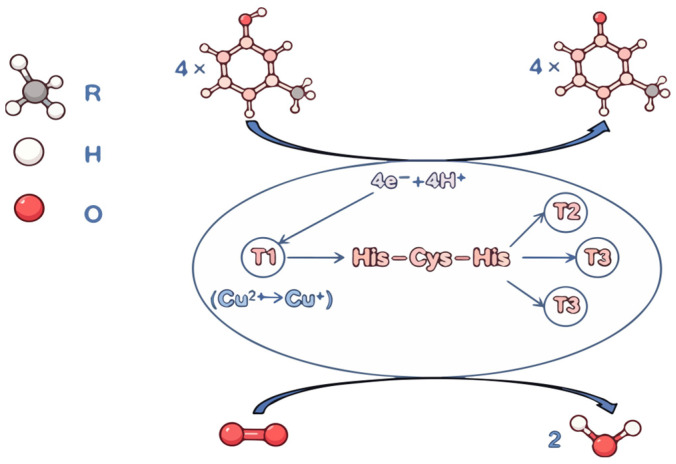
Schematic representation of the laccase catalytic cycle. Electrons are transferred from phenolic substrates to the surface-located copper center T1, then transferred to the trinuclear copper cluster T2/T3, where molecular oxygen is reduced to water (adapted from Petr Baldrian from *FEMS Microbiology Reviews*, 2006 [14]).

Laccase is a copper-rich protein, composed primarily of a peptide chain, sugar ligands, and Cu^2+^. The peptide chain typically contains 220 to 800 amino acids, with a molecular weight ranging from approximately 50 to 140 kDa [10]. Laccases from different sources exhibit relatively high sequence homology. Although laccase has been reported in bacteria, fungi, actinomycetes, and plants, it is most frequently documented in fungi [11]. Based on amino acid sequence evolution and functional commonality in catalysis, laccases are classified under the auxiliary activity family 1 (AA1) within the carbohydrate-active enzymes. The AA1 family comprises three subfamilies, with typical laccases belonging to the first subfamily (AA1-1) [12]. Certain small-molecule mediators—such as syringaldehyde, acetylsyringone, acetylvanillin, and p-coumaric acid—can effectively broaden the substrate range of laccase, enhancing its catalytic efficiency and stability [13]. The catalytic cycle of laccase revolves around its copper-containing active site. At the T1-type copper site, the substrate (e.g., phenolic compounds) loses electrons and is oxidized. These electrons are then transferred via the conserved His-Cys-His electron transfer chain to the trinuclear copper center, which consists of T2- and T3-type coppers. Simultaneously, at the trinuclear center, oxygen accepts four electrons and four protons and is reduced to water, thereby completing the catalytic cycle. This coupled oxidation of the substrate and reduction of oxygen constitutes the core mechanism underlying laccase-mediated reactions, including lignin degradation, environmental remediation, and other applications [14] (Figure 1). Numerous studies have demonstrated that laccase possesses strong stability and a broad substrate spectrum, endowing it with significant practical application value.

### 2.2. Function and Action of Laccase

#### 2.2.1. Catalytic Function of Laccase

Laccase possesses important biochemical properties, including a broad substrate spectrum and the ability to use molecular oxygen as the final electron acceptor. The initial electron acceptor in laccase-catalyzed oxidation is Cu T1, located in the cavity near the enzyme surface [15]. In the catalytic reaction of laccase, the reduction of Cu T1 represents a rate-limiting step. The relatively low redox potential of Cu T1 (ranging from 420 to 790 mV vs. normal hydrogen electrode, NHE) restricts laccase to substrates containing phenolic moieties [16]. Based on redox potential, laccases are classified into two types: low-redox-potential and high-redox-potential laccases. Low-redox-potential laccases are found in bacteria, plants, and insects, whereas high-redox-potential laccases are widely distributed in fungi [17].

Laccase catalyzes both anabolic and catabolic reactions. A typical catabolic process involves the degradation of lignin and humus by fungal laccase. In anabolic reactions, laccase participates in morphogenesis, such as the synthesis of polymeric pigments [18], stratum corneum hardening [19], polyflavonoid synthesis [20], lignification [21], and humification of soil organic matter [22]. During anabolic processes, the redox potential of laccase in plants, bacteria, and insects enables radical coupling reactions to proceed thermodynamically without the need for additional chemicals [23].

#### 2.2.2. The Role of Laccase in the Growth of Edible and Medicinal Fungi

Laccase is a core functional enzyme for the growth and development of edible fungi, primarily serving the following five roles:

(1) Core nutritional function: degradation of lignin and release of carbon sources

**Figure 2:**
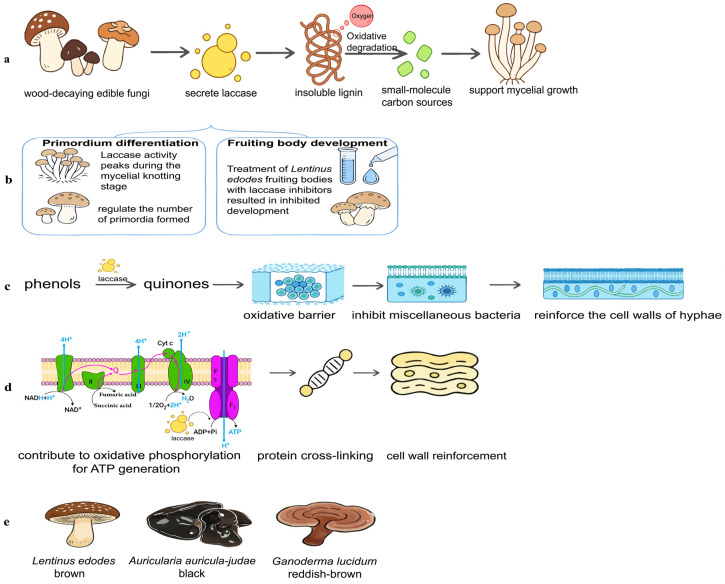
The role of laccase in the growth of edible fungi: (**a**) degradation of lignin and release of carbon sources, (**b**) primordium differentiation and fruiting body formation, (**c**) inhibition of contaminants and reduction in contamination, (**d**) involvement in respiration and substance synthesis, (**e**) pigment synthesis.

Laccase is a key extracellular enzyme involved in lignin degradation in wood-decaying edible fungi (such as *Lentinus edodes*, *Pleurotus ostreatus*, *Auricularia auricula-judae*, and *Ganoderma lucidum*). It oxidizes the phenolic and non-phenolic structures of lignin in the presence of oxygen, converting insoluble lignin into absorbable small-molecule carbon sources, thereby supporting mycelial growth, substrate utilization, and overall development [24] (Figure 2a).

(2) Developmental regulation: primordium differentiation and fruiting body formation

Primordium differentiation: Laccase activity peaks during the mycelial knotting stage, regulating the number of primordia formed, influencing fruiting density, and affecting fruiting body yield [25,26] (Figure 2b).

Fruiting body development: Treatment of *Lentinus edodes* fruiting bodies with laccase inhibitors resulted in inhibited development, confirming the important role of laccase in mushroom growth and maturation [27] (Figure 2b).

(3) Ecological defense: inhibition of contaminants and reduction in contamination

Laccase generates antimicrobial compounds such as quinones through the oxidation of phenols, forming an oxidative barrier that inhibits contaminants including Trichoderma, Penicillium, and bacteria, thereby reducing contamination rates during cultivation. Oxidation of phenolic compounds in the substrate to quinones directly suppresses contaminants, while also reinforcing the cell walls of hyphae to reduce pathogenic invasion [28] (Figure 2c).

(4) Energy and metabolism: involvement in respiration and substance synthesis

As an oxidase, laccase participates in the electron transport chain, contributing to oxidative phosphorylation for ATP generation to support mycelial growth and substance transport. It also catalyzes the transformation of phenolic and amine metabolites, playing a role in protein cross-linking and cell wall reinforcement [29] (Figure 2d).

(5) Other functions: pigment synthesis

Laccase is involved in the synthesis of cap pigments—such as the brown color of *Lentinus edodes*, the black color of *Auricularia auricula-judae*, and the reddish-brown color of *Ganoderma lucidum*—thereby influencing the appearance of fruiting bodies [1] (Figure 2e).

## 3. Cloning and Classification of Laccase Genes in Edible and Medicinal Fungi

Laccase, as a key member of the multicopper oxidase family, plays important roles in lignin degradation, pigment synthesis, and environmental pollutant treatment. Cloning laccase genes from edible and medicinal fungi is of great significance for the industrial advancement and ecological application. To date, researchers have successfully cloned multiple laccase genes using techniques such as transcriptome sequencing, RACE amplification, and RT-PCR, revealing interspecific differences in laccase genes that influence phenotypic traits. In terms of classification, laccase genes can be categorized into different subclasses based on sequence homology, enzymatic properties, and molecular mass. However, the current classification system still requires integration with multi-omics data to enhance its functionality. Additionally, cloning methods have room for improvement in terms of universality, efficiency, and optimization of heterologous expression.

### 3.1. Cloning of Laccase Genes in Edible and Medicinal Fungi

Laccase is an important member of the multicopper oxidase family. Cloning laccase genes from edible fungi is of great significance for further elucidating interspecific differences. Jin et al. employed gene knockout and overexpression techniques to demonstrate that the Lcc1 gene plays a key role in mycelial development and fruiting body maturation in *Ganoderma tsugae* [30]. Knockout of Lcc1 resulted in reduced lignin degradation efficiency, lighter fruiting body color, and shortened stipe length, whereas overexpression enhanced lignin degradation, indicating that Lcc1 is involved in cell wall remodeling and pigment synthesis. Fan et al. investigated the cloning, expression characteristics, and phylogenetic relationships of the laccase multigene family in *Auricularia auricula-judae* [31]. Using transcriptome sequencing, they successfully isolated seven lac genes and analyzed their expression patterns under different growth conditions (e.g., mycelium, primordium, and fruiting body) and developmental stages via quantitative reverse transcription PCR (qRT-PCR). Wu et al. cloned the full-length cDNA sequence using RACE technology and performed sequence analysis with bioinformatics tools. The modified cDNA fragment was inserted into the expression vector pPIC9K and transformed into *Pichia pastoris* GS115 for expression [32]. The recombinant protein was purified using a Ni column, and its laccase activity was detected using the ABTS substrate method. Niu Xin et al. found through RT-qPCR analysis that the expression level of Cc_lac21 was high during the mycelial stage and at the base of mature stipes, but low in the cap and the tips of elongated stipes [33]. They speculated that Cc_lac21 may be involved in substrate lignin degradation and mycelial cell wall maturation in stipes, laying a foundation for exploring the mechanism by which laccase genes participate in stipe cell wall maturation. Yang et al. first used RACE technology to clone the cDNA encoding the laccase lac1 gene and its full-length genomic sequence from the *Auricularia polytrica* AP4 strain [34] (Table 1).

Research on laccase gene cloning in edible and medicinal fungi has employed a variety of technical approaches, including gene knockout, overexpression, transcriptome sequencing, RACE cloning, and RT-qPCR. Some studies have combined morphological observations (such as fruiting body color and stipe length) with molecular mechanisms to link gene functions with phenotypic changes, enhancing the robustness of the conclusions. However, certain studies were conducted only under laboratory conditions without validation in natural environments, lacked in-depth exploration, or did not verify protein interactions or metabolic pathways.

### 3.2. Classification Basis of Laccase Genes Edible and Medicinal Fungi

Based on sequence homology, enzymatic properties, and molecular mass, laccase genes can be classified into distinct subcategories, each with different functions.

#### 3.2.1. Amino Acid Sequence Homology

**Figure 3:**
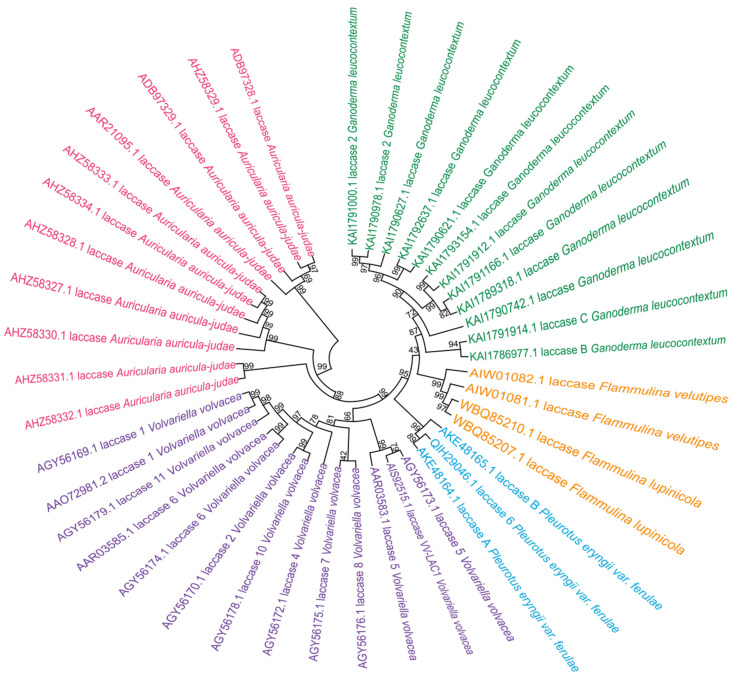
Phylogenetic tree of partial amino acid sequences of laccase. All sequences were taken from the NCBI database. The phylogenetic tree was built with the neighbor-joining (NJ) method using MEGA 11. Sequences are available in the Supplementary Material.

Laccases from different edible fungi differ in physicochemical properties, which are primarily dictated by their amino acid sequences. Although laccase sequences from different species show varying degrees of identity and clear divergence, most fungal genomes harbor multiple genes encoding laccase isozymes. For example, *Pleurotus eryngii var. ferulae* encodes 5 laccase sequences [35], *Ganoderma leucocontextum* has 14 [36], *Volvariella volvacea* contains 18 [37], and *Flammulina velutipes* has 13 [38]. Additionally, *Auricularia auricula-judae* harbors 11 [31], and *Pleurotus ostreatus* as many as 25 [39]. In this paper, laccase amino acid sequences from different types of edible fungi were retrieved from the NCBI database, and a phylogenetic tree was constructed (Figure 3). Variations in sequence length, conserved domains, and amino acid composition collectively contribute to the diversity of their physicochemical properties.

#### 3.2.2. Physicochemical Properties of Laccase

The properties of laccase vary across different sources and are primarily determined by their protein-coding genes. Optimal temperatures and pH values for laccase from different edible fungi also differ. Generally, the optimal temperature ranges from 25 to 80 °C, and the optimal pH ranges from 3.5 to 6.0, though notable variations exist among species. The optimal pH value of laccase from *Agaricus balchaschensis* is 2.2 and the optimal temperature is 40 °C [40]. Sun et al. found that the optimal temperature for laccase isolated from the *Agaricus placomyces* was 30 °C [41]. Hao et al. isolated a laccase from *Agaricus sinodeliciosus*. The optimal temperature was 50 °C and the optimal pH value was 5.0 [42]. Hu et al. isolated a laccase from *Agrocybe cylindracea* at an optimal temperature of 50 °C and an optimal pH range of 3 to 4 [43]. Ng et al. isolated a laccase from the fruiting bodies of *Cantharellus cibarius*, with an optimal pH value of 4 [44]. Kim et al. isolated a laccase from *Coprinus comatus*, with an optimal pH value of 4.3 and an optimal temperature of 25 °C [45]. Park et al. isolated a laccase from *Fomitopsis pinicola*, with an optimal temperature of 80 °C and an optimal pH value of 3.0 [46]. Si et al. isolated a laccase from *Ganoderma australe*, which had high activity and stability under the conditions of pH 5.0–8.0 and temperature 10–60 °C [47]. Huang et al. cloned the laccase gene lac1 from *Ganoderma fornicatum* and expressed it in *Pichia pastoris* [48]. The optimal temperature of the recombinant laccase was 55 °C, and it was stable within the pH range of 2.5 to 10.0. Nitheranont et al. cloned two laccase genes (Lac2 and Lac3) from *Grifola frondosa* and expressed them in *Pichia pastoris* [49]. Laccase Lac2 was stable at pH 6.0, and laccase Lac3 was stable within the pH range of 4.0 to 8.0. Wang et al. expressed the laccase gene of *Laccaria bicolor* in *Pichia pastoris* [50]. The optimal pH value of the recombinant laccase was 3.6, and the optimal temperature was 40 °C. Zhang et al. isolated a laccase from the fruiting bodies of *Lepiota ventriosospora*, with an optimal temperature of 60 °C and an optimal pH value of 4.0 [51]. Zhu et al. isolated a laccase from *Lepista nuda*, with an optimal pH value of 3.0 and an optimal temperature of 50 °C [52]. Zhou Jinyang isolated a laccase from the *Lepista sordida*, with an optimal pH value of 3.0 and an optimal temperature of 60 °C [53]. Zhang et al. expressed the laccase gene MiLacA of *Morchella importuna* in *Pichia pastoris* [54]. The optimal pH value of the recombinant laccase was 4, and the optimal temperature was 60 °C. Jeon et al. isolated a laccase from *Mycetinis scorodonius*, with an optimal temperature of 75 °C [55]. Zhang et al. studied the effect of the pH value of the culture medium on laccase of *Pholiota microspora*, and the optimal pH value of laccase of *Pholiota microspora* was 6.0 [56]. Wu et al. isolated a laccase from *Pleurotus cornucopiae*. The optimal pH value was 4.2 and the optimal temperature was 30 °C [57]. Bamigboye et al. isolated a laccase from *Pleurotus tuber-regium*, with an optimal pH value of 4 and an optimal temperature of 60 °C [58]. The optimal pH range of laccase from *Polyporus umbellatus* is 4.5 to 5.0, and the optimal temperature range is 45 to 50 °C [59]. Zhu et al. isolated a laccase from *Russula virescens*, with an optimal pH value of 2.2 and an optimal temperature of 60 °C [60]. Xu isolated a laccase from *Trametes robiniophila*, with an optimal pH of 2.2 and an optimal temperature range of 50 to 60 °C [61]. Xu et al. isolated a laccase from *Tricholoma matsutake*, with an optimal pH value of 5. The enzyme activity changed little within the temperature range of 20 to 80 °C [62]. Miao et al. isolated a laccase from *Tricholoma mongolicum*, with an optimal pH range of 2 to 3 and an optimal temperature of 30 °C [63]. The growth rate of *Wolfiporia coco* is positively correlated with laccase activity. Acidic conditions are conducive to the secretion of laccase by *Wolfiporia coco*. The laccase activity of *Wolfiporia coco* is the highest at 25 °C [64] (Table 2). In summary, laccase activity in most edible and medicinal fungi is optimal within a pH range of 2 to 6, typically peaking under acidic conditions. Fungal laccase exhibits its maximum catalytic rate for aromatic phenolic or amine substrates within the acidic pH range [65]. Phenolic compounds undergo acid–base transition (with a pKa typically in the neutral to alkaline range), and fungal laccase oxidizes these substrates in their protonated phenol form. This pH preference can be attributed to structural features near the active-site cavity: all known fungal laccases possess a conserved Asp (pKa 3.9) or Glu (pKa 4.1) residue at this site, which is believed to play a role in stabilizing the phenoxy radical intermediate formed during catalysis [66]. Consequently, the optimal pH for laccase activity lies close to or below the pKa of the phenolic substrate, i.e., within the acidic to neutral pH range. The optimal temperature of laccase depends on its overall structural stability, which is jointly modulated by multiple structural factors [67]. Species variation is a key factor contributing to differences in optimal temperature: fungal laccases are generally active within the range of 40–70 °C, whereas thermotolerant bacterial laccases remain active and stable even above 90 °C [68]. The temperature adaptability of fungal laccases is regulated at both the “species level” (significant differences among different species) and the “genotype level” (multiple laccase genes encoding distinct isoenzymes within the same species) [69]. Structural factors directly determine the optimal temperature. For example, glycosylation enhances thermal stability [70]; reduced flexibility of surface loops improves thermal stability [71]; and disulfide bonds and ionic interactions constitute the molecular basis for differences in thermal stability [72].

#### 3.2.3. Molecular Mass Difference

**Figure 4:**
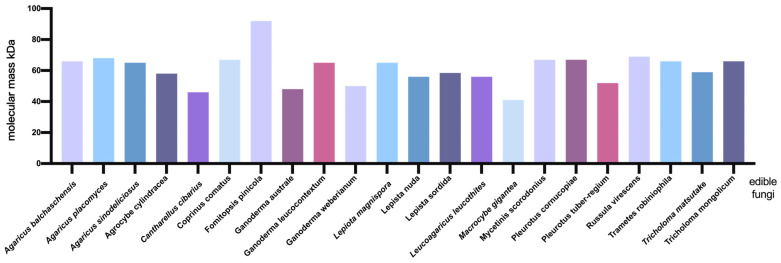
Molecular mass of laccase from different edible fungi.

The properties of laccase vary across different sources and are primarily determined by their protein-coding genes. The molecular masses of laccase in different edible fungi are not the same, and the molecular masses of laccase from different sources are also different. The molecular mass of laccase in *Agaricus balchaschensis* is 66 kDa [40]; Sun Jian et al. isolated laccase with a molecular mass of 68 kDa from *Agaricus placomyces* [41]. Hao Jingzhe et al. isolated a laccase with a molecular mass of 65 kDa from *Agaricus sinodeliciosus* [42]; Hu Dandan et al. isolated a laccase with a molecular mass of 58 kDa from *Agrocybe cylindracea* [43]. Ng et al. isolated a laccase from the fruiting body of *Cantharellus cibarius* [44]. This laccase was composed of two identical subunits, each with a molecular mass of 46 kDa. Kim et al. isolated a laccase with a molecular mass of 67 kDa from *Coprinus comatus* [45]. Park et al. isolated a laccase with a molecular mass of 92 kDa from *Fomitopsis pinicola* [46]. Si et al. isolated a laccase with a molecular mass of 48 kDa from *Ganoderma australe*, which had high activity and stability under the conditions of pH 5.0–8.0 and temperature 10–60 °C [47]. Umar et al. optimized the culture and nutritional conditions for laccase production in *Ganoderma leucocontextum*, and obtained laccase (with a molecular mass of 65 kDa) from *Ganoderma leucocontextum* through purification [73]. Zhou et al. cloned the laccase gene GwLac1 from *Ganoderma weberianum* and expressed it in *Pichia pastoris*, with a molecular mass of 50 kDa [74]; Rajagopalu et al. extracted laccase from *Hericium erinaceus*, with a molecular mass of 55 to 66 kDa [75]. Zhang et al. isolated a laccase with a molecular mass of 65 kDa from the fruiting bodies of *Lepiota magnispora* [51]. Zhu et al. isolated a laccase with a molecular mass of 56 kDa from *Lepista nuda* [52]. Zhou Jinyang isolated a laccase with a molecular mass of 58.5 kDa from the *Lepista sordida* [53]. Ning Yingjie et al. isolated a laccase with a molecular mass of 56 kDa from *Leucoagaricus leucothites* [76]. Rudakiya et al. isolated a laccase with a molecular mass of 41 kDa from *Tricholoma giganteum* [77]. Jeon et al. isolated a laccase with a molecular mass of 67 kDa from *Mycetinis scorodonius* [55]. Wu Xiangli et al. isolated a laccase with a molecular mass of 67 kDa from *Pleurotus cornucopiae* [57]. Bamigboye et al. isolated a laccase with a molecular mass of 52 kDa from *Pleurotus tuber-regium* [58]. Zhu et al. isolated a laccase with a molecular mass of 69 kDa from Russula virescens [60]; Xu Xin isolated a laccase with a molecular mass of 66 kDa from *Trametes robiniophila Murr* [61]. Xu et al. isolated a laccase with a molecular mass of 59 kDa from *Tricholoma matsutake* [62]. Miao et al. isolated a laccase with a molecular mass of 66 kDa from *Tricholoma mongolicum* [63] (Figure 4). The molecular weight of laccase is primarily determined by its protein-coding gene, degree of glycosylation, and subunit composition, all of which directly influence its structural stability, enzymatic properties, and application potential. Fungal glycosylation is widespread and often leads to an overestimation of molecular weight when measured by SDS-PAGE [78]. Deglycosylation can remove the excess sugar chains, thereby revealing the true molecular weight of the protein backbone. Following deglycosylation, the molecular weight decreases, and the altered glycosylation status can affect both the optimal pH and thermal stability of the enzyme. Conversely, the introduction of additional glycosylation sites has been shown to enhance specific activity and significantly improve thermal stability. Such glycosylation variants may confer an adaptive advantage by helping fungi cope with fluctuating microenvironmental conditions [79]. Furthermore, the complexity of subunit composition can also influence the overall molecular weight. In the absence of subunits, differences in molecular weight are primarily attributable to the degree of glycosylation and variations in the protein backbone itself [80].

## 4. Expression Regulation of Laccase in Edible and Medicinal Fungi

The expression of laccase in edible and medicinal fungi is influenced by various external factors, including carbon and nitrogen sources in the culture medium, aromatic compounds, metal ions, and other exogenous substances, as well as diverse chemical agents. Nutritional conditions—such as the carbon-to-nitrogen ratio, inorganic salts, and Cu^2+^ concentration—significantly affect laccase gene expression. Regulation by these external factors and inducers primarily occurs at the transcriptional level. The regulation of laccase gene expression varies considerably among different species of edible and medicinal fungi, and even within the same strain, expression levels differ substantially under varying induction conditions, primarily manifested as distinct laccase expression profiles.

### 4.1. The Influence of Nutritional Conditions on the Regulation of Laccase Expression in Edible and Medicinal Fungi

**Figure 5:**
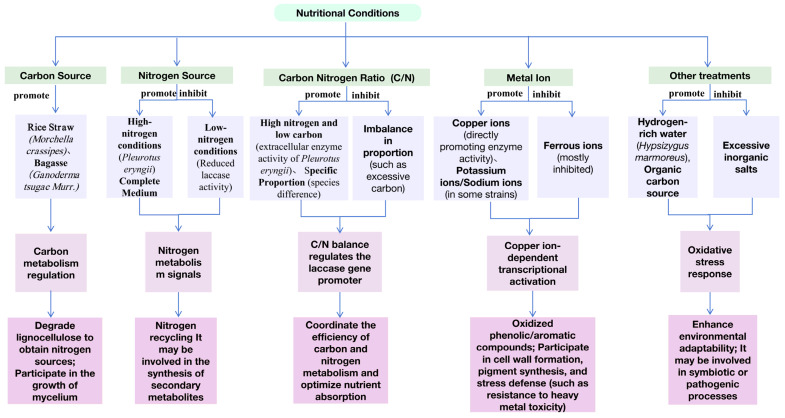
Schematic representation of the regulation of laccase expression by external factors, including carbon source, nitrogen source, carbon/nitrogen ratio, metal ions, and environmental conditions.

Most edible and medicinal fungi can secrete laccase, which plays a crucial role in their growth and development. Laccase activity determines the rate at which these fungi degrade nutrients in the culture medium, thereby affecting the efficiency of nutrient absorption and utilization. Carbon and nitrogen sources are two fundamental nutrients for the growth of most edible and medicinal fungi. They not only influence fungal growth and laccase secretion but also regulate the expression of laccase genes. Specifically, the type, composition, and ratio of carbon to nitrogen sources all affect laccase synthesis in edible and medicinal fungi. Chen et al. treated the mycelium of *Hypsizygus marmoreus* with hydrogen-rich water and found that this enhanced laccase activity in the mycelium [81]. KANWAL et al. reported that carbon sources significantly affected laccase production in *Morchella crassipes*, with rice straw increasing laccase activity to 12.6 U/mL [82]. Ai et al. found that extracellular laccase activity in *Pleurotus eryngii* was higher under high-nitrogen, low-carbon conditions, as well as in high-inorganic-salt conditions and complete media [83]. Low-nitrogen conditions resulted in decreased laccase activity. In contrast, laccase gene expression at the transcriptional level was elevated under low-nitrogen, complete, and high-nitrogen, low-carbon conditions. The addition of organic carbon sources such as bagasse promoted both laccase gene transcription and extracellular enzyme activity in *Ganoderma tsugae Murr*. Meanwhile, Cu^2+^ induced laccase secretion but had no effect on laccase gene transcription. Adjustments to the carbon-to-nitrogen ratio resulted in marked differences in laccase gene expression and extracellular enzyme activity between the two edible fungi, with high nitrogen levels promoting laccase secretion in the strains. Cui concluded that Cu^2+^ promotes laccase activity in *Agrocybe aegerita*. With the exception of K^+^, Zn^2+^, and Na^+^, which enhance the activity of certain laccase genes, other metal ions inhibit laccase activity. The study also explored the effects of different substrates and metal ions on the catalytic reaction of laccase [84]. Xiao found that the metal ion Fe^2+^ inhibits laccase activity in *Armillaria mellea*, while Mg^2+^ and Cu^2+^ promote it. The four ions Ca^2+^, K^+^, Na^+^, and Zn^2+^ have little effect on laccase activity [85]. Yang reported that at metal ion concentrations of 0.5 and 1.0 mmol/L, Cu^2+^ and Ni^2+^ promote the activity of *Ganoderma sinense* laccase Lac3, whereas the presence of Fe^2+^ completely inhibits its activity [86] (Figure 5). Previous studies have clarified the promoting effects of different carbon sources (such as rice straw and bagasse) and nitrogen sources on laccase activity, revealed the enhancing effect of Cu^2+^ on enzyme activity, and identified the inhibitory effects of ions such as Fe^2+^. These studies cover multiple species including *Hypsizygus marmoreus*, *Pleurotus eryngii*, and *Ganoderma tsugae Murr*. However, most studies have focused on single factors, with a lack of multi-factor interaction analysis (e.g., the synergistic effects between carbon-to-nitrogen ratio and metal ions). Additionally, research has primarily concentrated on changes in enzyme activity during short-term fermentation (e.g., 7–15 days), without tracking the stability or phenotypic adaptability of laccase genes during long-term culture.

### 4.2. The Expression Regulation Mechanism of Laccase Genes in Edible and Medicinal Fungi

Laccase breaks down chemical bonds in lignin, degrading it into small-molecule substances that can be absorbed and utilized by fungi. Niu et al. speculated that the Cc_lac21 gene in *Coprinus comatus* is highly expressed during the mycelial stage, providing nutrients for mycelial growth and facilitating lignin degradation in the culture medium. *Pleurotus ostreatus* is a common edible fungus and a typical white-rot fungus, known for its ability to effectively degrade lignin in crop straw [33]. Jiao conducted bioinformatics and gene expression pattern analyses of the laccase gene family in *Pleurotus ostreatus*, identified the candidate gene PoLac2 associated with lignin degradation, and constructed a eukaryotic overexpression vector for PoLac2, confirming its significant role in straw lignin degradation during the mycelial stage [87]. Liu reported that LCC8, LCC2, and LCC12 in *Ganoderma lucidum* are the primary laccase genes transcribed during the primordium formation, cap formation, and early sporulation stages, respectively. It is inferred that LCC8, LCC2, and LCC12 are involved in early lignin degradation, cap formation, and spore formation during the growth of *Ganoderma lucidum* [88]. Additionally, the expression of the LCC4 gene plays a significant role in laccase synthesis in *Ganoderma lucidum*.

Laccase plays a regulatory role during the fruiting body development stage. Niu Xin et al. speculated that Cc_lac21 is involved in the maturation and aging process of the stipe cell wall in fruiting bodies, providing a reference for further exploration of the mechanisms by which *Coprinus comatus* regulates fruiting body growth and development [33]. Laccase is involved in both lignin degradation and the growth and development of edible fungi. To elucidate the role of laccase genes in the growth and development of Pleurotus ostreatus, Zhuo Rui et al. found that lacc12 is highly expressed during primordium differentiation and fruiting body formation, indicating its involvement in the fruiting process. lacc4, lacc7, and lacc11 are highly expressed during the primordium differentiation stage and are associated with primordial cell differentiation. The expression levels of lacc2, lacc3, and lacc8 increase significantly at the mature fruiting body stage, suggesting their roles in fruiting body differentiation and maturation [89]. Wu et al. cloned the laccase-encoding gene fv-lac4 from *Flammulina velutipes* and found that its expression level was very low during the mycelial stage but became highly expressed beginning at the primordium stage of fruiting body formation. These results suggest that fv-lac4 is involved in fruiting body growth and development [90]. Li et al. determined the lignin content and laccase activity in the cultivation substrate at different growth and developmental stages of *Pleurotus eryngii*, and examined the expression of ten laccase family genes in the mycelium within the substrate using real-time fluorescence quantitative PCR. The results showed that during both the mycelial and fruiting body stages, the expression levels of Lac5 and Lac6 were significantly higher than those of the other genes [91] (Table 3).

## 5. Summary and Future Perspectives

This article systematically reviews the research progress on laccase in edible and medicinal fungi, covering its physiological functions, gene cloning, classification basis, and expression regulation mechanisms. As a key member of the multicopper oxidase family, laccase is structurally composed of copper-containing peptide chains, sugar ligands, and Cu^2+^. It exhibits excellent catalytic and redox capabilities, playing crucial roles in lignin degradation, pigment synthesis, and environmental pollutant treatment. In terms of gene cloning, researchers have successfully isolated and identified laccase genes from multiple species using techniques such as transcriptome sequencing, RACE amplification, and gene knockout. These studies have revealed species-specific differences in sequence homology, enzymatic properties (including optimal temperature/pH and molecular weight), and expression patterns. Regarding classification, laccase genes can be categorized into distinct subclasses based on sequence homology and functional differences. Studies on expression regulation have shown that the type and ratio of carbon and nitrogen sources, as well as metal ions such as Cu^2+^, significantly affect laccase activity and gene transcription levels. High nitrogen sources promote laccase secretion, Cu^2+^ enhances enzyme activity, while ions such as Fe^2+^ exhibit inhibitory effects. In terms of regulatory mechanisms, laccase genes display stage-specific expression during mycelial growth, fruiting body development, and lignin degradation. However, the underlying regulatory networks still require validation through protein interaction and metabolic pathway analyses. Future research may focus on multi-factor interaction analysis, long-term stability tracking, in-depth molecular mechanism studies (such as using CRISPR/Cas9 to verify transcription factor interactions), cross-species comparisons and functional exploration (e.g., screening for thermotolerant laccases), as well as environmental adaptability investigations (e.g., simulating natural substrate culture systems). Such efforts will further refine the theoretical framework of laccase gene regulation and accelerate its translational applications in fields such as biodegradation, medical health, and carbon neutrality.

## Figures and Tables

**Table 1:** Cloning Information of Laccase Genes from Representative Edible and Medicinal Fungi.

Species	Laccase Gene	Technological Means	References
*Ganoderma tsugae*	Lcc1	qRT-PCR	[30]
*Auricularia auricula-judae*	lcc1~7	qRT-PCR	[31]
*Volvariella volvacea*	vv-lac1, vv-lac6	RACE	[32]
*Coprinus comatus*	Cc_lac21	RT-qPCR	[33]
*Auricularia polytricha AP4*	lac1	RACE	[34]

**Table 2:** The physiological properties of laccase in representative edible and medicinal fungi.

Scheme	Optimum Temperature	Optimum pH	References
*Agaricus balchaschensis*	40 °C	2.2	[40]
*Agaricus placomyces*	30 °C	—	[41]
*Agaricus sinodeliciosus*	50 °C	5.0	[42]
*Agrocybe cylindracea*	50 °C	3.0~4.0	[43]
*Cantharellus cibarius*	—	4	[44]
*Coprinus comatus*	25 °C	4.3	[45]
*Fomitopsis pinicola*	80 °C	3.0	[46]
*Ganoderma australe*	10~60 °C	5.0~8.0	[47]
*Ganoderma fornicatum*	55 °C	2.5~10.0	[48]
*Grifola frondosa*	—	Lac2 6.0, Lac3 4.0~8.0	[49]
*Laccaria bicolor*	40 °C	3.6	[50]
*Lepiota magnispora*	60 °C	4.0	[51]
*Lepista nuda*	50 °C	3.0	[52]
*Lepista sordida*	60 °C	3.0	[53]
*Morchella importuna*	60 °C	4	[54]
*Mycetinis scorodonius*	75 °C	—	[55]
*Pholiota microspora*	—	6.0	[56]
*Pleurotus cornucopiae*	30 °C	4.2	[57]
*Pleurotus tuber-regium*	60 °C	4.0	[58]
*Polyporus umbellatus*	45~50 °C	4.5~5.0	[59]
*Russula virescens*	60 °C	2.2	[60]
*Trametes robiniophila*	50~60 °C	2.2	[61]
*Tricholoma matsutake*	20~80 °C	5.0	[62]
*Tricholoma mongolicum*	30 °C	2.0~3.0	[63]
*Wolfiporia coco*	25 °C	pH < 7	[64]

**Table 3:** The expression regulation mechanisms of laccase genes.

Species	Gene	Expression Pattern	Functional Specificity	References
*Coprinus comatus*	Cc_lac21	High expression during the mycelial growth period	Lignin degradation	[33]
*Pleurotus ostreatus*	PoLac2	High expression during the mycelium stage	Lignin degradation	[87]
*Ganoderma lucidum*	LCC8	High expression in the early growth stage	Lignin degradation	[88]
*Coprinus comatus*	Cc_lac21	The fruiting body is highly expressed during the growth period	The cell wall of the fruiting body stalk matures and ages	[33]
*Pleurotus ostreatus*	lacc12	The differentiation of primordia and the formation period of fruiting bodies are highly expressed	Promote mushroom formation	[89]
*Pleurotus ostreatus*	lacc4, lacc7, lacc11	High expression during the primordial differentiation period	Differentiation of primordia	[89]
*Pleurotus ostreatus*	lacc2, lacc3, lacc8	The expression level significantly increased at the mature fruiting entity stage	The differentiation and maturation of fruiting entities	[89]
*Flammulina velutipes*	fv-lac4	The formation of primordia of fruiting entities began to show high expression	The growth and development of entities	[90]
*Pleurotus eryngii*	Lac5, Lac6	High expression in the mycelium stage and fruiting body stage	Promote mushroom formation	[91]

## Data Availability

No new data were created or analyzed in this study. Data sharing is not applicable to this article.

## Supplementary Materials

The following supporting information can be downloaded at https://www.mdpi.com/article/10.3390/jof12050350/s1.
