# Supplementary material for: Research Progress of Laccase in Edible and Medicinal Fungi
Source: J Fungi (Basel). 2026 May 8;12(5):350. doi: 10.3390/jof12050350 (PMC13208001; doi:10.3390/jof12050350)
Supplement: Supplementary file 1 [file jof-12-00350-s001.zip › jof-4279525-supplementary.pdf]

>QIH29046.1 laccase 6 [*Pleurotus eryngii var. ferulae*]
MAVAFIALVSLALALVRVEASIGPRGTLNIANKVIQPDGFSRSAVLAGGSYPGPLIKGKTGDRFQINVVNE
LADTSMPVDTSIHWHGLFVKGHNWADGPAMVTQCPIVPGHSFLYDFEVPDQAGTFWYHSHLGTQYCDG
LRGPFVVYSKNDPHKRLYDVDDESTVLTVGDWYHAPSLSLAGVPHPDSTLFNGLGRSLNGPASPLYVMN
VVKGKRYRIRLINTSCDSNYQFSIDGHTFTVIEADGENTQPLQVDQVQIFAGQRYSLVLNANQAVGNYWI
RANPNSGDPGFANQMNSAILRYRGARNVDPRTPEKNATNPLREYNLRPLIKEPAPGKPFPGGADHNINLN
FAFDPATALFTANNYTFVPPTVPVLLQILSGTHDAHDLAPAGSIYDIKLGDVVEVTMPALVFAGPHPMHL
HGHSFAVVRSAGSSTYNYENPVRRDVVSIGDDPTDNVTIRFVADNAGPWFLHCHIDWHLDLGFAVVFAE
GVNQTAVANPVPEAWNDLCPIYNSSNPSKLLMGTNAIGRLPAPLKA

>AKE48165.1 laccase B [*Pleurotus eryngii var. ferulae*]
MAVAFIALVSLALALVRVEASIGPRGTLNIANKVIQPDGFSRSAVLAGGSYPGPLIKGKTGDRFQINVVNE
LADTSMPVDTSIHWHGLFVKGHNWADGPAMVTQCPIVPGHSFLYGFEVPDQAGTFWYHSHLGTQYCDG
LRGPFVVYSKNDPHKRLYDVDDESTVLTVGDWYHAPSLSLAGVPHPDSTLFNGLGRSLNGPASPLYVMN
VVKGKRYRIRLINTSCDSNYQFSIDGHTFTVIEADGENTQPLQVDQVQIFAGQRYSLVLNANQAVGNYWI
RANPNSGDPGFANQMNSAILRYRGARNVDPRTPEKNATNPLREYNLRPRIKEPAPGKPFPGGADHNINLN
FAFDPATALFTANDYTFVPPTVPVLLQILSGTHDAHDLAPAGSIYDIKLGDVVEVTMPALVFAGPHPMHL
HGHSFAVVRSAGSSTYNYENPVRRDVVSIGDDPTDNVTIRFVADNAGPWFLHCHIDWHLDLGFAVVFAE
GVNQTAVANPVPEAWNDLCPIYNSSNPSKLLMGTNAIGRLPAPLKA

>AKE48164.1 laccase A [*Pleurotus eryngii var. ferulae*]
MAVAFIALVSVALALVRVEASIGPRGTLNIANKVIQPDGFSRSAVLAGGSYPGPLIKGKTGDRFQINVVNE
LADTSMPVDTSIHWHGLFVKGHNWADGPAMVTQCPIVPGHSFLYDFEVPDQAGTFWYHSHLGTQYCDG
LRGPFVVYSKNDPHKRLYDVDDESTVLTVGDWYHAPSLSLAGVPHPDSTLFNGLGRSLNGPASPLYVMN
VVKGKRYRIRLINTSCDSNYQFSIDGHTFTVIEADGENTQPLQVDQVQIFAGQRYSLVLNANQAVGNYWI
RANPNSGDPGFANQMNSAILRYRGARNVDPRTPEKNATNPLREYNLRPLIKEPAPGKPFPGGADHNINLN
FAFDPATALFTANNYTFVPPTVPVLLQILSGTHDAHDLAPAGSIYDIKLGDVVEVTMPALVFAGPHPMHL
HGHSFAVVRSAGSSTYNYENPVRRDVVSIGDDPTDNVTIRFVADNAGPWFLHCHIDWHLDLGFAVVFAE
GVNQTAVANPVPEAWNDLCPIYNSSNPSKLLMGTNAIGRLYAPLKA

>KAI1793154.1 laccase [*Ganoderma leucocontextum*]
MAKFQSLLSCTTLLFAASAYAGIGPKTDLTISNANIAPDGYTRAAVVVNGVFPSPLITGNKGDRFQLNVID
QMTNHTMLKSTSIHWHGFFQKGTNWADGPAFVNQCPISSGHSFLYDFQVPDQAGTYWYHSHLSTQYCD
GLRGPFVVYDPSDPLKKLYDVDDDSTVITLSDWYHVAARLGPRFPLGSDSTIINGLGRSTSNATAELAVIS
VTQGKRYRFRLVSLSCDPNYTFSVDDHDMTVIEADGIETQPVTVNAIQIFSAQRYSFVLTANQTIDNYWIR
ANPSFGNIGFTDGINSAILRYAGADSVEPVTSQQTTQNLLNEVDLHPFTAKRTPGLPTPGGVDQAINMVFN
FNGSSFFINNASFTPPTVPVLLQILSGAQAAQDLLPSGSVYTLPINKSIELTFPATANAPGAPHPFHLHGHAF
AVVRSAGSTVYNYDNPVWRDVVSTGTPAAGDNVTIRFQTDNPGPWFLHCHIDFHLDAGFAVVFAEDAP
DVSLANPVPKAWSDLCPTYDALSADDQ

>KAI1792637.1 laccase [*Ganoderma leucocontextum*]
MSRGSPIALLSALSLSLTSALAAIATTADLDLTTSGIPVDVALGAQVGTTAELTISNADIAPDGYTRAAIVV
NGQHPGPLLTGNKVRPRHAFALLFRLASIQRSQGDTFQVNVIDQLTNGTMLKSTSVHFHGVDQIGTNWE
DGAAFVNQCPIATGHSFTYEFSCIDQAGTFWYHSHLSSQYCDGLRGPMIVYDPNDPHADLYDVDDDSTII

TLADWYHTATRVNTKLQFGPDSVLVNGLGRYAGGDSTDLAVISVTQGKRYRFRLISMSCDPNFTFSIDGH
NMTIIEVDAINHEPLAVDSIQIFAGQRYSFVLTANQAIDNYWVRALPNSGTTNFDGGVNSAILRYVGATET
EPTTTQTNSTAPLTETDLVPLNHAAAPGDPISGGVDYALNLVMSFNGTKFFMNGATFTPPTAPVLLQILSG
ASSATDLLPSGSVYTLPSNATVELSFPVTSANRVGAPHPFHLHGHTFSVIRSAGSESYNYANPPRRDVVNT
GTAGDNVTIRFRTDNPGPWFLHCHIDPHAEAGLGVVLAVDTNTTSTFTTTDAWKDLCPTYNALSSDDL

>KAI1791912.1 laccase [*Ganoderma leucocontextum*]
MARFQSLLSYITLLCAASAYAGIGPQTDLTISNADIAPDGFTRAAVVVNGVFPGPLITGNMGDNFQLNVV
DQLTNHTMLKTTSIHWHGFFQKGTNWADGPAFVNQCPIASGNSFLYDFQVPDQAGTFWYHSHLSTQYC
DGLRGPFVVVYDPNDPLADLYDVDDDSTVITLADWYHTAARLGPAFPFGSDSTLINGLGRSPATSTANLTV
ISVTQGKRYRFRLVSLSCDPNYTFSIDGHDLTVIEADGIETQSVTVNAIQIFAAQRYSFVLTANQTIDNYWI
RANPSFGNVGFTDGINSAILRYDGADTIDPVTSQQTTQNLLAETDLHPLVAKQTPGNPTQGGVDMAINMV
FNFNGTKFSINGATFVPPTVPVLLQILSGAQTAQSLLPSGSVYALPLNSSIELTFPATANAPGAPHPFHL
HGHAFAVVRSAGSTVYNYDNPVWRDVVSTGTPAAGDNVTIRFQTDNPGPWFLHCHIDFHLEAGFAVVF
AEDAPDTSADNAVPQAWSDLCPTYDALSSDDQ

>KAI1791166.1 laccase [*Ganoderma leucocontextum*]
MARLQSLLPYFTLLFAASAYAAIGPTADLTITDADIAPDGFTRAAVVVNGVFPAPLITGNKASPGDRFQLN
VINQLTNHTMLKTTSIHWHGIFQEGTNWADGPAFVTQCPIASGDSFLYDFRVPDQAGTFWYHSHLSTQYC
DGLRGPLVVYDPHDPLAHLYDVDDDSTVITLTEWYHFAAKLGPRFPAGMANSTLINGLGRSTDTPTAEL
AVVNVTQGKRYRFRLVSMSCDPNFTFSIDGHNLTVIEADSIETQPVTVNTIQIFTAQRYSFVLTANQTIDNY
WIRANPNFGVVGFADGLNSAILRYAGAAPIDPVTSQQSTTVLLNETDLHPYVPRKTPGKPTKGGVDLALN
MAFGFNTTDYFINNATFVPPSVPVLLQILSGTQTAQDLLPAGSVYTLPKNSSIEITFPANANAAGSPHPFHL
HGHAFAVVRSAGSTAYNYHNPVWRDTVSMGLASVADNVTIRFQTDNPGPWFLHCHIDFHLAAGFAVVL
AEDVPDIAYANPVPQAWSNLCPTYDALSPDDQ

>KAI1790742.1 laccase [*Ganoderma leucocontextum*]
MAVFQFLHASAFVALKLALTAHGAIGPVTDLTITNANIAPDGYTRPAVLADGILPGPLISGQKGDHFQINVI
NNLSNHTMLKSTSIHWHGLVQKSTNWADGGASVNQCPIATGNSFLYEFDTQDQAGTFWYHSHVSTQYC
DGLRGALVVYDPSDPHQDLYDVDNDDTVITLADWYHTTYTLGPKPLQGAQSTLINGKGRSTTTLAADLA
VISVTQGRRYRFRLVSTSCDVNFVFSIDHHPVTVIEADAVNTRPHTVDSIHIFAGQRYSFVLTANQTVDNY
WIRAKPNLGYLSFESGMNSAILRYEGAPETEPTPSFPPASVKPLVETALRPLVPMPVPGTAEIGGVDLAINL
VITFNKSHFFVNNKTFVPPPVPVLLQIMSGAKDAKDLLPSGSVFVLPSNANVQFSFPIAGHLAGVPHP
IHMHGHEFAVVRSAGSSEYNYKDPIWRDVVSTGTTGDNVTIRFRTDNPGPWFLHCHIDPHIEEGLAIVMV
EGKSSAIQANNSPPEAWDDLCPTYNALSVSDQ

>KAI1790627.1 laccase [*Ganoderma leucocontextum*]
MSKFSSFAGLIALSFSLSYTRLVAGAIGPTADLTISNAVISPDGFPRAAIVMNGVFPGPLITGNKGANFQINV
VDDLTNATMLTATTVHWHGLFQKGTNWADGPSMVNQCPISMGNSFLYDFTATGQAGTFWYHSHLSTQ
YCDGLRGPLVVYDPDDPHASLYDVDDESTVITLTDWYHIAARLGPRFPPGADSVLINGLGRFTDGNSTEL
AVINVTQGKRYRFRLLSLSCDPNFMFSIDNHNMTVIEADAVNHQALTVDEIQIFAGQRYSFVLTANQAVD
NYWIRALPNIGTTSFAGGINSAILRYSGADDIEPTTTQTGGSNLLVESRLVPLTDLAAPGTAKPGGVDYAL
NMAFSFNGTEFFINGTPFVSPSVPVLLQILSGTTNAADLLPKGSIYALPANASIELSFPITAINAPGAPHPFHL

HGHTFHVVRSAGQTDYNYVDPPQRDVVSTGTAGDNVTIRFTTNNPGPWFLHCHIDFHLEAGFAVVFAED
TPDVAATETPTAAWENLCPIYASAYPTGL

>KAI1790621.1 laccase [*Ganoderma leucocontextum*]
MSRGSLIALLSALSLSLTSALAAIATTADLDLTTSGIPVDVALGAQVGTTAELTISNADIAPDGYTRAAIVV
NGQHPGPLLTGNKGDTFQVNVIDQLTNGTMLKSTSVHFHGVDQIGTNWEDGAAFVNQCPIATGHSFTYE
FSCIDQAGTFWYHSHLSSQYCDGLRGPMIVYDPNDPHADLYDVDDDSTIITLADWYHTATRVNTKLQFG
PDSVLINGLGRYAGGDSTELAVISVTQGKRYRFRLISMSCDPNFTFSIDGHNMTIIEVDAINHEPLAVDSIQI
FAGQRYSFVLTANQAIDNYWVRALPNSGTTNFDGGVNSAILRYVGATETEPTTTQTNSTAPLTETDLVPL
NHAAAPGDPISGGVDYALNLVMSFNGTKFFMNGATFTTPTAPVLLQILSGASSATDLLPSGSVYTLPSNAT
VELSFPVTSANRVGAPHPFHLHGHTFSVIRSASESYNYANPPRRDVVNTGTAGDNVTIRFRTDNPGPWFL
HCHIDPHAEAGLGVVLAVDTNTTSTFTTTDAWKDLCPTYNALSSDDL

>KAI1789318.1 laccase [*Ganoderma leucocontextum*]
MPARLGSLLLYLTVLFTASAFAAIGPIADLIISNADVAPDGFTRAAVVVNGVFPSSLITGNKGDHFRLNVIN
EMTNHTMLKTTSIHWHGLFQKGTNWADGPTFVTQCPITSGDSFLYDFRVPGQAGTFWYHSHLSTQYCDG
LRGPLVVYDPFDPLAWLYDVDDDSTVITLADWYHFAAKLGPRFPAGLANSTLINGLGRSPDTPAADLAVI
NVTHGNRYRFRLVSLSCDPNYTFSIDGHNLTVIEADGIETHPVVVNAIQIFAAQRYSFVLTANQAVDNYWI
RANPNLGIIGFEGGLNSAILRYDGANSIEPETSLQSTQDLLKETDLHPLFPRKTPGRPKQGGVDLALNMAF
NFNGTNFFINNATFDPPPVPVLLQILSGAQTAQDLLPPGDVYALPPHSSIEITFPATARAPGGPHPFHMHGH
AFAVVRSAGSTAYNYHNPVWRDTVSTGTSDANDNVTIRFRTDNPGPWFLHCHIDFHLDAGFAVVLAEDI
PRTPCLMRGHGCARLMTRSRRRTIKRTCLTRASTDGSDKDIGWDIVM

>KAI1791914.1 laccase C [*Ganoderma leucocontextum*]
MAKGNSYTAVAVLVLATIAPPSLAAIGPVTDLAIVNRPVSPDGIPRNAVLAGGTFPGPVVSGYSGDHFKIN
VKDELYNTTMLTGTSIHWHGLLQHTTNWADGAAFINQCPITSGNSFEYKFDTTGISGTYWYHSHLGNQY
CDGLRGPLVLYDKNDPHKHLYDVDDDSTVLTLADWYHVASPFVLTRGGPPRADSTLINGLGRWYENPT
AELAVIKVTHGKRYRFRLINIACDPNYNFTIAGHSMTIIEADGQNSEPLVVDELQIFVAQRYSFVLEANQPI
DNYWIRALPDVMANNGSLGYVNGINSAILRYEGAPAEEPCEQEVKSMNPLHEYNLHSLTDPAAPGDPHV
GGVDYALNLALGFNNASKTPFNINGVPFQSPSVPVLLQILSGARKAQDLLPGGSVYTLPRNATIEVTIPPLA
VGGPHPFHLHGHAFSVVRSSGQAAPNYVNPIKRDVVSTGFGNDNVTIRFRTDNPGPWLLHCHIDWHLSG
GLAIVFAEDVPDVSFVDAAPSEQIFSP

>KAI1791000.1 laccase 2 [*Ganoderma leucocontextum*]
MAKAGTFAALAAFSLPYARLVAGAIGPIADLTIADADITPDGFTRAAVVMNGQFPGPLITGNKGDNFQLN
VVNDLDNTTMLTATTVHWHGFFQQGTNWADGVAMISQCPISADNSFLYDFTATDQAGTFWYHSHLSTQ
YCDGLRGPMVVYDPDDPHASLYDVDDETTVITLSDWYHTAARLGNAFPGGADSTLVNGLGRFAGGDST
DLAVFNVTQGQRYRFRLVAMSCDPNFTFSIDGHNMTVIEADAINHAAVVVDEIQIFAGQRYSFVLTADQD
VDNYWIRALPNIGTTTFDGGVNSAILRYAGADAIEPTTNQTTSVLALNETDLVPLENLVPPGTAEVGGVD
YALNLNFGFNGSEFFINDVAFVPPSVPALLQILSGTTAAASLLPSGSYYSLPSNSSIEISFTMVAADAPGAPH
PFHLHGHSFYVVRSAGETTYNYVNPPQRDVVSVGADGDNVTIRFTTDNPGPWFLHCHIDFHLEAGFAIVL
AEETDEVTTSVTPSSAWEDLCPIYASAYPSGLTKRKRHAHRRSLDASF

>KAI1790978.1 laccase 2 [*Ganoderma leucocontextum*]

MAKARTFAALAAFSLPYARLVAGAIGPIADLTIADADITPDGFTRAAVVMNGQFPGPLITGNKGDNFQLN
VVNDLDNTTMLTATTVHWHGFFQQGTNWADGVAMISQCPISADNSFLYDFTATDQAGTFWYHSHLSTQ
YCDGLRGPMVVYDPDDPHASLYDVDDETTVITLSDWYHTAARLGNAFPGGADSTLVNGLGRFAGGDST
DLAVFNVTQGQRYRFRLVAMSCDPNFTFSIDGHNMTVIEADAINHAAVVVDEIQIFAGQRYSFVLTADQD
VDNYWIRALPNIGTTTFDGGVNSAILRYAGADAIEPTTNQTTSVLALNETDLVPLENLVPPGTAEVGGVD
YALNLNFGFNGSEFFINDVAFVPPSVPALLQILSGTTAAASLLPSGSYYSLPSNSSIEISFTMVAADAPGAPH
PFHLHGHSFYVVRSAGETTYNYVNPPQRDVVSVGADGDNVTIRFTTDNPGPWFLHCHIDFHLEAGFAIVL
AEETDEVTTSVTPSSAWEDLCPIYASAYPSGLTKRKRHAHRRSLDASF

>KAI1786977.1 laccase B [*Ganoderma leucocontextum*]
MARDRTLLRNVFALLGATRVALGAIGPVTDLAIVNKVIAPDGFARDTVLAGGTFPGPLIQGHKGDRFRVN
VIDQLQNETMLTATSIHWHGLFQHTTNWADGPAFVTQCPITKDHSFLYDFRTPDQAGTFWYHSHLSTQY
CDGLRGPLVVYDPYDPQAHLYDVDNEDTVITLADWYHLAATVVQSRPGAPTPNSVLINGLGRWTGNPTS
ELAVINVEHGKRYRFRVVSMSCDPSFNFSIDNHSLTVIEADGQSTKPLNVDSLVILAAQRYSVVLTANQTV
GNYWIRANPNNNEGKGFANGINSAILRYKGAPEQEPNSAQTPSLRPLVETGLQPLSNPAAPGRPYPGGVD
HALHLNLGFSPPNFTINDAKFDPPNLPVLLQILSGNRTAQSLLPKGSVYTLPPYSTIELSFNVSAPGAPHPFH
LHGHAFSVVRSAGSAEYNYNNPVRRDTVSTGVGTDNVTIRFVTDNPGPWFLHCHIDWHLQAGLAIVFAE
DTKDTALVNPVPESWKELCPTYYGANQTHY

>AAO72981.2 laccase 1 [*Volvariella volvacea*]
MQSLLNTLTAMVTVVGWGANFAAAALSSHTLTLTNGFASPDGHSREVVLVNGGLFQAVIAGNKGDDFEI
EVDNQLTVEILRKSTSIHWHGLFQRGSAWADGPAFVTQCPIAPGNTFTYEFTPTDEVGTFWYHSHLDAQY
CDGLRGPFVIYDPNDPHLALYDVDDEDTIITLADWYHTAAELLTGVVIPDSALINGLGRTSTTTTSPLAVIN
VVQGTKYRMRLISISCDPNNYIFSIEDHEMTVIEADGISVDPVTVTSLQIFVGQRYSFILHANQPVDNYWIR
ANPNLGPTGFSGGINSAILRYQGAPVADPTGPGLQDALNRLAETDLHPIVNPGAPDPVDIDLVINIGFSAGL
FNIDGTSYVSPDVPSLLQILNGVPVSSLLPSGSYIELPPNKVVQLSFPVIAGQGSAIGAPHPIHLHGHAFDVIR
SAGSSTYNLVDPVRRDVVSIGTPGDNVTIRFVTDNAGPWYLHCHIEWHLRAGLGVIFAEDAAGSLLQPAP
PTAWGDLCDIYDLLPPEDQ

>AGY56178.1 laccase 10 [*Volvariella volvacea*]
MAFSSRMQSLLNALTFMITLMTLGVSYAAAQQSSHTLELTNAQVTPDGRQREAILVNGGLFGAVITGQK
GDEFVIEVDNQLSSSLLRKSTSIHWHGLFQRDSAWADGPAFVTQCPIAPGHTFTYRFTATDEVGTFWYHS
HLDAQYCDGLRGPFIIYDPNDPHLSLYDVDNEDTIITLADWYHTPAEQLSGGISPQSTLINGQGRSSANFNA
PLSVVNVQQGLKYRMRLISMSCDPNWIFSIEGHELTVIEADGISVQPVTVTSLQIFVGQRYSFVLHANQPV
GNYWIRANPNKGPTGFGNNINSAILRYQGAPIADPTGTGLQDSLNRLNEPDLHPVVNPGAPGLAQIDGADI
DLVVNIGFSGGRFTIDGTSYTSPDVPSLLQILSGTPPSSLLPTGSYIELPANKVVQISFPVISGQGSAIGAPHP
MHLHGHAFDVIRSAGSPVYNYVDPPRRDVVAIGRPGDNVTIRFVTDNAGPWYLHCHIEWHLRSGLGVIIA
EDSQGVINGPQPPPEWEELCDIYNALDSEDK

>AGY56179.1 laccase 11 [*Volvariella volvacea*]
MVTVMGWGAGFTAAALSSHTLTLTNGVRSPDGHPREVVLVNNDLFQTVITGDKGDDFEIEVDNQLTVEI
LRKSTSIHWHGLFQRGSAWADGPAFVTQCPIAPGNTFTYEFTPTDEVGTFWYHSHLDAQYCDGLRGPFVI
YDPNDPHIALYTVDNEDTIITLADWYHDPAAELSGVINPDSTLINGLGRTGTTTTSPLAVINVVQNTKYRM

RLISISCDPNFIFSIEDHELTIIEADGISVTPVTVTSLQIFVGQRYSFVLHANQPVGNYWIRANPNKGPTGFVD
GINSAILRYQGAANADPTGPGLQDSLNRLQETDLHPIVNPGAPGPDDINGADVDLVINIGFAAGAFNIDGT
TYVSPDVPSLLQILSHGDPSLLMPSGSYIELPPNKVVQLSFPVIAGQGSAIGAPHPIHLHGHVFDVIRSAGSS
TYNFDDPPRRDVVSIGSPGDNVTIRFVTDNAGPWYLHCHIEWHLRSGLGVILAEDVQGTFTGPAPPSAWD
DLCDIYDQLPPEDQ

>AGY56176.1 laccase 8 [*Volvariella volvacea*]
MSLSLQFQSLLNTVTIMMIVMGLGVVAQQSVHTLELTNGPVSPDGVTRQAVLVNGGLFSKVITGQKGDE
FVIEVDNQLTLPELRKSTSIHWHGLFQRGSQWADGPAFVTQCPISPGHKFTYRFTATEEAGTYWYHSHLD
AQYCDGLRGAFVIYDPNDPHLDLYDVDDESTVITLADCNPDATLINGLGRTSATPTAPLAVVNVQKDVK
YRFRLVSISCDPNWVFSIEGHELTVIEADGVSMQPVIVTSLQILAGQRYSFVLHANQPVDNYWIRANPNLP
PTGFNSNINSAILRYQGAPEQEPTSSGIQDTSKPLLETNLHPVVNPGAPGLDNIDGADVPIVLPIGLASGRFN
INGTSLQLPDVPTLLQILSGANAVEDLLPSEHYIQLPPNKVIQISFPVIAGQGSAIGGPHPIHLHGHTFDVIRS
AGNSTYNYVDPPRRDVVSMGGPGDNVTIRFVTDNAGPWFLHCHIEWHLQAGLGVVLAENIPGVASSPQP
PQEWEELCNAYNSLAPEDQ

>AGY56175.1 laccase 7 [*Volvariella volvacea*]
MVTVVGLGVDHASAQQSSVTLELTNGAVSPDGITRDAILVNGALFGAVITGKKGDHFVIEVDNQLNNTD
LRKSTSIHWHGLFQRGSSWADGPAFVTQCPIAPDHKFTYQFTPTEEAGTFWYHSHLDAQYCDGLRGPFIIY
DPEDPHLDLYDVDNEDTIITLADWYHTPAPQLSGIITPDSTLINGLGRTVATPTAPLAVINVQQGLRYRFRL
LSLSCDPNWVFSIEGHDLTVVEADGVSMQPVTVSSIQIFAAQRYSFILNANQPVDNYWIRANPNIGPTGFN
NNINSAILRYQGAPVAEPTGLGVQDTLDLLFEPDLHPVVNPGAPGVAQSNGVDIDLVISIAFTSGRFFLDGT
SYTSPDMPSLLQILSGTPASDLLSPGSYIPLPANKTIQISFPVIAGQGSAVGAPHPFHLHGHTFDVVRSAGSS
VVYNYIDPPRRDVVAIGLPGDNVTIRFVTDNAGPWFLHCHIEWHLRNGLGVVIAEDTAGVVNGAAPPQEW
EELCDIYNALDPEDQ

>AGY56174.1 laccase 6 [*Volvariella volvacea*]
MALSLRIQSLLNTLTVVLTVMGLGVGFVAAGTASHTLTLTLTNGVVSPDGHSREVVLVNGALFGKLITGRK
GDDFEIEVDNQLTNSLLRKSTSIHWHGLFQRGSAWADGPAFVTQCPIAPGNTFTYSFTPTDEVGTFWYHS
HLDAQYCDGLRGPFVIYDRFDPHRHRYDVDNKDTIITLADWYHTPAAQLSGVITPNSALINGRGRTATTT
TAPLSVIRVRRGRKYRMRLISMSCDPNYIFSIEGHEMTVIEADGISVEPVTVTSLQIFVGQRYSFVLHANQP
VGNYWIRANPNKGPTGFVDGINSAILRYHGAPNAEPTGAGLQDALNRLAEPDLHPIVNPGAPGLATPDGV
DIDIVVNIGFAAGLFNIDGTSYVSPDVPTLLQILNGVPVGSLMPSGSYIALPANKVVQISFPVVSGQGSAIGA
PHPIHLHGHAFDVVRSAGSATYNYADPPRRDVVSIGTPGDNVTIRFVTDNAGPWYLHCHIEWHLRAGLG
VILAEDAPGVLTGPAPPSEWDDLCDIYDGLDEEDQ

>AGY56173.1 laccase 5 [*Volvariella volvacea*]
MAYSARLGSALSTLAIMLLVLALGVGHATAQASPPQSVHTLNLTNANVSPDGYNRSAILVNGNLLNEAIV
GNKGDQFVIMVENNLDNPLLRKSTSIHWHGLFQRGTQWADGPAFVTQCPIAPEHSFTYQFTAGHEAGTF
WYHSHLDAQYCDGLRGPFVIYDPADPHLSLYDVDDNSTIITLADWYHEPASQLSGIVIPKSTLINGLGRTN
TTTDAPLAVINVEQGVKYRFRLVALSCDPNWVFSIEGHDLTVIESDGISVQPVTVTSLQIFAGQRYSFVLH
ANQAVDNYWIRANPNLGPTGFADGINSAILRYNGAPDEEPTGSGLQDPSNRLQEPDLHPVANPGAPGFDE
IDGVDVNLVINIGFAAGRFSMAGASFEPPSLPALLQILSGTTDVADLLPAGSYIELPANKVVQISFPVISGGG

AATGAPHPIHLHGHTFDVIRSAGSSTYNFVDPPRRDVVSIGTPGDNVTIRFVTDNAGPWFLHCHIEWHLRS
GLGVILAEDIPGIVSGPQPPGTFIPSHDVGTYVRLWDLLGTTAAWEELCDIYNDLDEEDR

>AGY56172.1 laccase 4 [*Volvariella volvacea*]
MLFFSSRVKYLLGHLAVITAVVGGDVGNPGDHSGGYPGGHSEDHHGGPSGGHSGSHSDHSGSHRSFHTL
KLTNGRVSPDGFQRNAVLVNGGLFQTAITGNKGDEFAIKIDNKLTNSLSHKSTSIHWHGLFQHGTPWADG
PAFVSQCPIAPGHQYTYRFSSADQAGTFWYHSHLDAQYCDGLRGPFIIYDPDDPHRSLYDIDNKDTIITLA
DWYHRDSNLLSGIVIPNSTLINGLGRTAQSPNTPLAVVNVKQGLRYRMRLVAISCDPNWIFSIEGHKLTVI
EADGVSVQPVTVTSLQIFVAQRYSFVLHANQPVGNYWIRANPNIGPTGFDSNINSAILRYKGAPIAEPRGL
GLQNSDNRLREPDLHPLFNPGAPGLAHVDGADINMVINIGFIQSAGRFTIDGTSYVPPDVPTLLQILSGTPA
SSLLPSGSYIALPPNKVVQLSFPVFDGQGSALGGPHPIHLHGHTFDVIRSAGSSEYNFVNPPRRDVVAIGTP
GDNVTIRFATDNAGPWYLHCHIEWHLRAGLGVVIAEDAPGLANGPEPPEEWKQLCPIYNALDPDDK

>AGY56170.1 laccase 2 [*Volvariella volvacea*]
MAFSSSRMQSLLNTLTVMITLMGLGVSYAAAQQSFHTLELTNDEVNPDGFQRDAVLVNGGLFGAVITGQ
KGDEFVIEVDNQLTDSLLRKSTSIHWHGLFQRDSAWADGPAFVTQCPIAPGHTFTYRFTATEEAGTFWYH
SHLDAQYCDGLRGPFIIYDPNDPHLGLYDVDNEDTIITLADWYHTPASPSSCRLSSIYPSFYSSPQSTLINGL
GRSSSDFTAPLAVVNVQQGLKYRMRLISISCDPNWIFSIEGHELTVIEADGISVQPVTVTSLQIFVGQRYSFV
LHANQPVGNYWIRANPNKGPTGFGNNINSAILRYQGAPIADPTGAGLQDALNRLAEPNLHPLVNPGAPGL
AQINGADIDIVINIGFSGGLFNIAGTSYTSPDVPSLLQILSGTPPSSLMPSGSYIELPGNKVVQLSFPVIAGQGS
AIGAPHPIHLHGHAFDVIRSAGSNVYNYVDPPRRDVVSIGRAGDNVTIRFVTDNAGPWYLHCHIEWHLRS
GLGVIIAEDSQGVVNGPQPPPEWEELCDIYDALIPEDQ

>AGY56169.1 laccase 1 [*Volvariella volvacea*]
MVTVVGWGANFAAAALSSHTLTLTNGFASPDGHSREVVLVNGGLFQAVIAGNKGDDFEIEVDNQLTVEI
LRKSTSIHWHGLFQRGSAWADGPAFVTQCPIAPGNTFTYEFTPTDEVGTFWYHSHLDAQYCDGLRGPFVI
YDPNDPHLALYDVDDEDTIITLADWYHTAAELLTGVVIPDSALINGLGRTSTTTTSPLAVINVVQGTKYR
MRLISISCDPNYIFSIEDHEMTVIEADGISVDPVTVTSLQIFVGQRYSFILHANQPVDNYWIRANPNLGPTGF
SGGINSAILRYQGAPVADPTGPGLQDALNRLAETDLHPIVNPGAPDPVDIDLVINIGFSAGLFNIDGTSYVS
PDVPSLLQILNGVPVSSLLPSGSYIELPPNKVVQLSFPVIAGQGSAIGAPHPIHLHGHAFDVIRSAGSSTYNL
VDPVRRDVVSIGTPGDNVTIRFVTDNAGPWYLHCHIEWHLRAGLGVIFAEDTAGSLLQPAPPTAWGDLC
DIYDLLPPEDQ

>AAR03585.1 laccase 6 [*Volvariella volvacea*]
MGLGVGFVAAGTASHTLTLTNGVVSPDGHSREVVLVNGALFGKLITGRKGDDFEIEVDNQLTNSLLRKS
TSIHWHGLFQRGSAWADGPAFVTQCPIAPGNTFTYSFTPTDEVGTFWYHSHLDAQYCDGLRGPFVIYDRF
DPHRHRYDVDNKDTIITLADWYHTPAAQLSGVITPNSALINGRGRTATTTTAPLSVIRVRRGRKYRMRLIS
MSCDPNYIFSIEGHEMTVIEADGISVEPVTVTSLQIFVGQRYSFVLHANQPVGNYWIRANPNKGPTGFVDG
INSAILRYHGAPNAEPTGAGLQDALNRLAEPDLHPIVNPGAPGLATPDGVDIDIVVNIGFAAGLFNIDGTSY
VSPDIPTLLQILNGVPVGSLMPSGSYIALPANKVVQISFPVVSGQGSAIGAPHPIHLHGHAFDVVRSAGSAT
YNYADPPRRDVVSIGTPGDNVTIRFVTDNAGPWYLHCHIEWHLRAGLGVILAEDAPGVLTGPAPPSEWD
DLCDIYDGLDEEDQ

>AAR03583.1 laccase 5 [*Volvariella volvacea*]

MAYSARLGSALSTLAIMLLVLALGVGHATAQASPPQSVHTLNLTNANVSPDGYNRSAILVNGNLLNEAIV
GNKGDQFVITVENNLDNPLLRKSASIHWHGLFQRGTQWADGPAFVTQCPIAPEHSFTYQFTAGHEAGTF
WYHSHLDAQYCDGLRGPFIIYDPADPHLSLYGVDDNSTIITLADWYHEPASQLSGIVIPKSTLINGLGRANT
TTDAPLAVIHVEQGVKYRFRPVALSCDPNWVFSIEGHELTVIESDGVSVQPVTVTSLQIFAGQRYSFVLHA
NQAVDNYWIRANPNLGPTGFADGINSAILRYNGAPDEEPTGSGLQDPSNRLHEPDLHPVANPGAPGFDEI
DGVDVNLVINIGFAAGRFSMAGASFEPPSLPALLQILSGTTDVADLLPAGSYIELPANKVVQISFPVISGGG
AATGAPHPIHLHGHTFDVIRSAGSSTYNFVDPPRRDVVSIGTPGDNVTIRFVTDNAGPWFLHCHIEWHLRS
GLGVILAEDIPGIVSGPQPPAAWEELCDIYNDLDEEDR

>AIS92515.1 laccase VV-LAC1 [*Volvariella volvacea*]
MAYSARLGSALSTLAIMLLVLALGVGHATAQASPPQSVHTLNLTNANVSPDGYNRSAILVNGNLLNEAIV
GNKGDQFVIMVENNLDNPLLRKSTSIHWHGLFQRGTQWADGPAFVTQCPIAPEHSFTYQFTAGHEAGTF
WYHSHLDAQYCDGLRGPFVIYDPADPHLSLYDVDDNSTIITLADWYHEPASQLSGIVIPKSTLINGLGRTN
TTTDAPLAVINVEQGVKYRFRLVALSCDPNWVFSIEGHDLTVIESDGISVQPVTVTSLQIFAGQRYSFVLH
ANQAVDNYWIRANPNLGPTGFADGINSAILRYNGAPDEEPTGSGLQDPSNRLQEPDLHPVANPGAPGFDE
IDGVDVNLVINIGFAAGRFSMAGASFEPPSLPALLQILSGTTDVADLLPAGSYIELPANKVVQISFPVISGGG
AATGAPHPIHLHGHTFDVIRSAGSSTYNFVDPPRRDVVSIGTPGDNVTIRFVTDNAGPWFLHCHIEWHLRS
GLGVILAEDIPGIVSGPQPPAAWEELCDIYNDLDEEDR

>AIW01082.1 laccase [*Flammulina velutipes*]
MLRALLTSTTLSLLVSRALAAIGPVADLVIANADVSPDGFTRTAALAGGTVSGVIITGNKGDNFQINVVNS
LNDSRILQSTAIHWHGMFMAGTNWADGPAFVSQCPIAKGNSFLYDFTALDQAGTYWYHSHLSTQYCDGI
RGPLIIYDPDDPHASLYDVDNDDTVITLADWYHVFARGQAGVPTADAILINGLGRWASDPTSELAVINVQ
FGVRYRFRLVSMSCDPNFLFSIQGHTLNVIEADGVSIVPRETDQIQIFAGQRYSFVLTADQEIANYWIRAIP
NLPAPPANPVENGINSAILRYVTADVAEPLDDSIDIRNPLDENALVPLENPGAPTGTIFPVNLQFGFNPANL
QFTVNGAPFIPPDVPVLLQLLSGAQTPDTLMPGGSIIRLPANAVVELSMPSGGLAGSPHPFHLHGHVFDVIR
GPGQPANTEPNLTNPPRRDTVSIGNAGDNVTVRFVTDNPGPWFLHCHIDWHLELGLAVVFAEDTDRWVS
NIDPSPAWDELCPIYSALPPGDL

>AIW01081.1 laccase [*Flammulina velutipes*]
MSRSLTAFITLSLLVLRSFAGIGPITDLVIANADVAPDGFTRTAALSGGTVNGALITGSRGDNFQINVVNNL
NDNNILQSTSIHWHGMFMAGTSWADGPAFVNQCPIAKSNSFVYDFTALEQAGTFWYHSHLSSQYCDGIR
GPMVIYDPDDPHAALYDVDNDDTVITLADWYHAFAKTLAFPTPDSTLINGLGRWAEDPTSDLAVINVEA
GVRYRFRLVSMSCDPNFVFAIQGHTLNIIEADGVSTEPIEVDQITIFAGQRYSFVLTADQAVDNYWIRAEPT
PGPTGFEDGINSAILRYSGAAEAEPTDDTVESTNPLDENALVPLENPGADGTAEVGADDVFAINLALAFDL
STFKFTVNGATFEPPTTPVLLQLLSGAQTADTLLPAGSLFTLPANRVVELSIPSEGLIGGPHPFHLHGHVFD
VIRGPGQTDYNFENPPRRDVVSIGAAGDNVTIRFTTDNPGPWFLHCHIDWHLEAGLALVFAEDTDNWDV
STPPTSWDELCPIYDALSEDDL

>WBQ85210.1 laccase [*Flammulina lupinicola*]
MSRSLTAFITLSFLVLRAFAGIGPITDLVIANADVAPDGFTRTAALSGGTVNGALITGSRGDNFQINVVINL
NDNNILQSTSIHWHGMFMAGTSWADGPAFVNQCPIAKSNSFVYDFTALEQAGTFWYHSHLSSQYCDGVR
GPMVIYDPDDPHAALYDVDNDDTVITLADWYHAFAKTLAFPTPDSTLINGLGRWAEDPTSDLAVINVEA
GVRYRFRLVSMSCDPNYVFAIQGHTLNIIEADGVSTEPIEVDQITIFAGQRYSFVLTADQAVDNYWIRAEP

TPGPTGFEDGINSAILRYSGADEAEPTDDTVESTNPLDENALVPLENPGADGTAEVGADDVFAINLALAFD
LSTFKFSVNGATFEPPTTPVLLQLLSGAQTADTLLPSGSLFTLPANRVVELSMPSEGLIGGPHPFHLHGHVF
DVIRGPGQTDYNFENPPRRDVVSIGAAGDNVTIRFTTDNPGPWFLHCHIDWHLEAGLALVFAEDTDNWD
VSTPPTSWDELCPIYDALSEDDL

>WBQ85207.1 laccase [*Flammulina lupinicola*]
MSRSLTAFITLSFLVLRAFAGIGPITDLVIANADVAPDGFTRTAALSGGTVNGALITGSRGDNFQINVVNNL
NDNNILQSTSIHWHGMFMAGTSWADGPAFVNQCPIAKSNSFVYDFTALEQAGTFWYHSHLSSQYCDGVR
GPMVIYDPDDPHAALYDVDNDDTVITLADWYHAFAKTLAFPTPDSTLINGLGRWAEDPTSDLAVINVEA
GVRYRFRLVSMSCDPNYVFAIQGHTLNIIEADGVSTEPIEVDQITIFAGQRYSFVLTADQAVDNYYIAGIRA
EPTPGPTGFEDGINSAILRYSGADEAEPTDDTVESTNPLDENALVPLENPGADGTAEVGADDVFAINLALA
FDLSTFKFSVNGATFEPPTTPVLLQLLSGAQTADTLLPSGSLFTLPANRVVELSMPSEGLIGGPHPFHLHGH
VFDVIRGPGQTDYNFENPPRRDVVSIGAAGDNVTIRFTTDNPGPWFLHCHIDWHLEAGLALVFAEDTDN
WDVSTPPTSWDELCPIYDALSEDDL

>AHZ58334.1 laccase [*Auricularia auricula-judae*]
MRLSLAILAAVAPSLASILPPLVHTRAPHRSISHRQTCTNDDSRLCWDGTYDISTNYYNDGPDTGKTVVY
YLQITNTTLAPDGVSRQVLTINGTIPGPTITANWGDRLEIHVSNAMKDNGTSIHWHGIRQLQNSINDGVNG
VTECPLAPGQTKVYSFKATQYGTSWYHSHYSAQYGDGVWGAIVINGPATAEYDVDLGPITINEWFHQTA
YAAAYAAERSGPPRPQNFLIGGTNALATDTTKGKRFTRTFESGKKYRLRLINTSVDSFFRVSLDNHTMTVI
ASDFVPIKPYTTNVIGLAIGQRYDVIIEANQDATKSYWFRVWPQISCSTNDNAGDVTGYITYGTTDLPTST
AFEVDDSCDDETGLVPYVSVTVDSSGFSAGETDISVSGPSRVTVQGENVFRWLVNGAAMDVDWSYPTL
QQVADNNDTFAAVQNALFFDEGLSTAYWVIQNAGGVAHPIHLHGHDFNVISTGLGTFDASTATLQWENP
PRRDVAMLPGGGYLFIAFETDNPGVWVMHCHIAWHVSEGLSVQLVERQSEILDTVTLDDAWEDTCSTW
KSWYSGDLFWGKKTDSGL

>AHZ58333.1 laccase [*Auricularia auricula-judae*]
MRLSLAILAAVAPSLASILPPLVHTRAPHRSISHRQTCTNDDSRLCWDGTYDISTNYYNDGPDTGKTVVY
YLQITNTTLAPDGVSRQVLTINGTIPGPTITANWGDRLEIHVSNAMKDNGTSIHWHGIRQLQNSINDGVNG
VTECPLAPGQTKVYSFKATQYGTSWYHSHYSAQYGDGVWGAIVINGPATAEYDVDLGPITINEWFHQTA
YAAAYAAERSGPPRPQNFLIGGTNALATDTTKGKRFTRTFESGKKYRLRLINTSVDSFFRVSLDNHTMTVI
ASDFVPIKPYTTNVIGLAIGQRYDVIIEANQDATKSYWFRVWPQISCSTNDNAGDVTGYITYGTTDLPTST
AFEVDDSCDDETGLVPYVSVTVDSSGFSAGETDISVSGPSRVTVQGENVFRWLVNGAAMDVDWSYPTL
QQVADNNDTFAAVQNALFFDEGLSTAYWVIQNAGGVAHPIHLHGHDFNVISTGLGTFDASTATLQWENP
PRRDVAMLPGGGYLFIAFETDNPGVWVMHCHIAWHVSEGLSVQLVERQSEILDTVTLDDAWEDTCSTW
KSWYSGDLFWGKKTDSGL

>AHZ58332.1 laccase [*Auricularia auricula-judae*]
MALLSLVLLAAAATPGLGALVEKWWDITFVNANPDGLFERRVVGVNGSWPPPPIELTVNDTLRVHATNK
LDKPTTLHHHGMFFNKTSYFDGALGVSQCGIPPGQTFTYDVDVAGSGQWGTYWVHAHASGHYVDGLR
APVVIHNIPEAHDYDDEFTVVLGDWYHDQHADLLKKFISRGNPGGAEPVPDSALIYFSHNGAYLRSSTNP
NAVGFSENATLPFEPGKTYRLRIVNTSAFSMFFFWIDGHDMRVIEVDGTDVEEFPASLLSLTVAQRYSVLV
TARNDTSSNFLIHANMDTDMFDVVPDALNPNTTATVVYDKNAKTADGVDKDAYFDFPDISLVPTIKEPQ
QPATHQIVLNVLFDTMTDGTNRAMFNNITYNPPVVPSIFSELSLGKDAVQAQLYGPTSFVLNQFDVVEIKV

VNFDAGKHPFHLHGHKFQIVHKSTDFTSDDPVLNPPLVEGQANPVRRDTVQVPSMGSATLRFVADNPGA
WLFHCHIEWHLEAGLAVTLFESPLEAQKILKPPQFMFDQCAALGVPTVGNAAGHTGSDLDGLKLGPFQQ
KLGWRPKGIVAMTGCVLTAVLGMATVAWYAWGGHISDEEVEEEVRRRQAAKSARPSIFSRMRGKN

>AHZ58331.1 laccase [*Auricularia auricula-judae*]
MALLSLVLLAAAATPGLGALVEKWWDITFVNANPDGLFERRVVGVNGTWPPPPIELTVNDTLRVHATNK
LDKPTTLHHHGMFFNKTSYFDGALGVSQCGIPPGQTFTYDVDVAGSGQWGTYWVHAHASGHYVDGLR
APVVIHNIPEAHDYDDEFTVVLGDWYHDQHADLLKKFISRGNPGGAEPVPDSALIYFSHNGVYLRSSTNP
NAVGFSENATLPFEPGKTYRLRIVNTSAFSMFFFWIDGHDMRVIEVDGTDVEEFPASLLSLTVAQRYSVLV
TARNDTSSNFLIHANMDTDMFDVVPDALNPNTTATVVYDKNAKTADGVDKDAYFDFPDISLVPTIKEPQ
QPATHQIVLNVLFDTMTDGTNRAMFNNITYNPPVVPSIFSELSLGKDAVQAQLYGPTSFVLNQFDVVEIKV
VNFDAGKHPFHLHGHKFQIVHKSTDFTSDDPVLNPPLVEGQANPVRRDTVQVPSMGSATLRFVADNPGA
WLFHCHIEWHLEAGLAVTLFESPLEAQKILKPPQFMFDQCAALGVPTVGNAAGHTGSDLDGLKLGPFQQ
KLGWRPKGIVAMTGCVLTAVLGMATVAWYAWGGHISDEEVEEEVRRRQAAKSARPSIFSRMRGKN

>AHZ58330.1 laccase [*Auricularia auricula-judae*]
MRSSNSVIVFFSALLTSCLALSIAPQPRSIAPQPDHYPTKCTNDQSRLCWDGKYDINTNYYTSGPRTGKTV
RYNLELKNVTLSPDGIPTQFLTINGQIPGPTLYANWGDTIEVHVTNKMHDNGTSIHWHGIRQLHTNKYDG
ANGVTECPLAPGDTKVYRFVAEQYGTSWYHSHYSVQYGDGAWGAIHINGPATSEYDIDLGTITLSEIYNQ
TAFQTGFISQTIEPPQPIPARNILINGTNVNVQNPSLGKRFQTTFTPGKKHRLRFINTAADSVFVVSLDSHNM
TVIQSDFVPIHPYTTDSISIAIGQRYDVIIDANQPVASYWLRTVVQTNCGETNDNTPELNLTGFIAYKGSKG
LPTTNGPLPPTNQDCHDETQLVPYLPIQVPKTQLSSSDQSVVPMNVSGPVSDVNDLGDTVTRWRIHGASID
VDWTYPTLQQLKDHNRTKPSNPIFLDKAGYTYWIIENQFIANHPIHLHGHDFNIIYQSGVNAGPFDPNSAV
LNFNNPPRRDVATLAGNGSLVIAFNLDNPGAWLLHCHIAWHVSQGLSVQLIERQQDIFKYTTLDNAWEK
TCTNFKKWYPTSIYGPKDDSGI

>AHZ58329.1 laccase [*Auricularia auricula-judae*]
MRLPVLLGALCAAGMASAALVEYDLVISQGNIKNDGQTKRSWLINGQSPGPAIVATKGDTVNVRVLNLG
SENITIQYVLIEQFLTPWSDGVPGLTQFPIRPLATFTYNFKVTQTGAYWYHSHHHMQMVDGLQGPVFLKP
DAQTVSSLTTLGLPEADQKKALHAELNPRFLSVFDNQHKSGEYVLSEWHKTGIEQLCLDDILINGKGQVV
CPDLDAIASIIPPAVGKVTKRGCAFPDNANLQPYGGKPSEVDPDLWFNCVPTSTPLEVFQVSASEKFVAFN
IVNSGTTWELRVSIDSHSFWVFTADGAYISPVKVNVISIPVGERFQILVPLDQSPGDYTIRVAAKAMPQLLS
GYAVLSYKKSLLNIKLGGVTSAPPAKHPFIDYGGNVISGSGGVLFNAMAIQPFPAQAPPSGPADVTIRISLQ
RSDALTWTMNGVPLPTLPEQFTPLLIDPKGISNLDPALYTSFKNGSIVDIILETTVLGKGSHPAHPIHKHNTK
AFFLGTGTGRFPAATVWEAFQRNTTGLNLVNPPLRDDFNTPPTKENTNWAALRFQSVNPMVTIMHCHID
PHLAGGMSFIMMEGLEALQPIPAYYKDFH

>AHZ58328.1 laccase [*Auricularia auricula-judae*]
MRLSLAILAAVAPALASLVPPLVHTRAPHRSLHRRQTCTNDDSRLCWDGTYDISTNYYDDGPDTGKTVV
YYLQITNTTLAPDGFSRQVLTINGTIPGPTLTANWGDRLEIHVTNALKDNGTSIHWHGIRQLQNTIADGVN
GVTECPLAPGQTKVYSFKATQYGTSWYHSHYSAQYGDGVVGAIVINGPASDEYDEDLGPITVSDWFHIT
AYAGAYAAERTGPPVPPNFLIGGTNALPTDTTKGKRFVKTIESGKKYRLRLINTSVDSFFRVSIDNHPMTVI
ASDFVPIKPYTTNVLGMAIGQRYDVIIEANQDTSKSYWFRVWPQISCSQNDNAGDVTGYITYGTTDLPTT
DAYDYDDSCDDEVGLEPYVSVTVDSSGFATSTQEDISVSAPTQVVVQGDNVFRWLVSGHAMDVDWGYP

TLQQVADNNNSYADIQNALFMDEGLSTAYWVIQNLGGIAHPIHLHGHDFNIVSSGSGTFDASTATLKWD
NPPRRDVAMLPASGYLFIAFQTDNPGVWVMHCHIAWHVSEGLSLQLVERQSEIFDTVTLDDEWQDTCST
WKTWYSGDLYWGKKTDSGL

>AHZ58327.1 laccase [*Auricularia auricula-judae*]
MLLSRTLTLIAGFVPAFASQLVDVRSSAALEARQTKPGCTNAQSRLCWDGKFDINTNYYTSGPRTGKKRV
YYWELRNTTLSPDGTPRLALAINGTMPGPTLIADWGDTIEVHLKNSLKDNGTSFHWHGIRQFENSKNDGV
NGVTECPLAPGDTKVYTFIAEQYGTSWYHSHYSAQYGDGVWGAIVINGPATAEYDIDLGTVTLSEWFRP
STAFEAALAAERTGPPRPQNFLQNGTNAMTEDVTKGKRFKTTFTPGKKHRLRFINTSVDTFFKVSLDGHN
MTVIQSDFVPIKPYTTNVIGLAIGQRYDVIIEANQPKASYWLRTWPQISCSANDNDGSVTGYVAYSGVTAL
PTSAATAFTDSCTDETQLVPQVPITVDSSGFPGNQSTIPVSAPQRVSIDGDMVFRWFINGAMMNIDFNYP
TLQQLADGNSTWNPPTNPIFLDKANTFAYWVIQNGSPVAHPIHLHGHDFNIVSSGTGNFSTSTATLKWDN
PPRRDVAMLPGNGYLFIAFKTDNPGAWLMHCHIAWHVSEGLSLQLIERQSEIFDHMTLDADWERTCDKF
ETWYDPATLAYGKKTDSGLRKRRVY

>AAR21095.1 laccase [*Auricularia auricula-judae*]
MRLPVLLGALCAAGMASAALVEYDLVISQGNIKNDGQTKRSWLINGQSPGPAIVATKGDTVNVRVLNLG
SENITIHWHGFFQPGPTVFPDSRSTLVCPLAPITFHLRVTQTGAYWYHSHHHMQMVDGLQGPVFLKPDAQ
TVSSLTTLGLPEADQKKALHAELHPRFLSVFDNQHKSGAGRLPGPRRDCVDIPPAVGKVTKRGCALPDNA
NLQPYGGKPSEVDPDLWFNCVPTSTPLEVFQVSASEKFVAFNIVNSGTTWELRVSIDSHSFWVFTADGAYI
SPVKVNVISIPVGERFQILVPLDQSPGDYTIRVAAKAMPQLLSGYAVLSYKKSLLNIKLGGVTSAPPAKHPF
IDYGGNVISGSGGVLFNAMAIQPFPAQAPPSGPADVTIRISLQRSHALTWTMNGVPLPTLPEQFTPLLIDPK
GISNLDPALYTSFKNGSIVDIILETTVLGKGSHPAHPIHKHNTKAFFLGTGTGRFPAATVWEAFQRNTTGLN
LVNPPLRDDFNTPPTKENTNWAALRFQSVNPMVTIMHCHIDPHLAGGMSFIMWRASRRYSPFRLTTRTFT
EPTHTHSLFLSNRTSSD

>ADB97329.1 laccase [*Auricularia auricula-judae*]
MRLPVLLGALCAAGMASAALVEYDLVISQGNIKNDGQTKRSWLINGQSPGPAIVATKGDTVNVRVLNLG
SENITIHWHGIEQFLTPWSDGVPGLTQFPIRPLATFTYNFKVTQTGAYWYHSHHHMQMVDGLQGPVFLKP
DAQTVSSLTTLGLPEADQKKALHAELHPRFLSVFDNQHKSGEYVLSEWHKTGIEQLCLDDILINGKGQVV
CPDLDAIASIIPPAVGKVTKRGCAFPDNANLQPYGGKPSEVDPDLWFNCVPTSTPLEVFQVSASEKFVAFN
IVNSGTTWELRVSIDSHSFWVFTADGAYISPVKVNVIRIPVGERFQILVPLDQSPGDYTIRVAAKAMPQLLS
GYAVLSYKKSLLNIKLGGVTSAPPAKHPFIDYGGNVISGSGGVLFNAMAIQPFPAQAPPSGPADVTIR
ISLQRSDALTWTMNGVPLPTLPEQFTPLLIDPKGISNLDPALYTSFKNGSIVDIILETTVLGKGSHPAHPIHK
HNTKAFFLGTGTGRFPAATVWEAFQRNTTGLNLVNPPLRDDFNTPPTKENTNWAALRFQSVNPMVTIMH
CHIDPHLAGGMSFIMMEGLEALQPIPAYYKDFH

>ADB97328.1 laccase [*Auricularia auricula-judae*]
MRLPVLLGALCAAGMASAALVEYDLVISQGNIKNDGQTKRSWLINGQSPGPAIVATKGDTVNVRVLNLG
SENITIHWHGIEQFLTPWSGGVPGLTQFPIRPLATFTYNFKVTQTGVYWYHSHHHMQMVDGLQGPVFLKP
DAQTVSSLTTLGLPEADQKKALHAELHPRFLSVFDNQHKSGEYVLSEWHKAGIEQLCLDDILINGKGQVV
CPDLDAIASIIPPAVGKVTKRGCAFPDNANLQPYGGKPSEVDPDLWFNCVPTSTPLEVFQVSASEKFVAFN
IVNSGTTWELRVSIDSHSFWVFTADGAYISPVKVNVISIPVGERFQILVPLDQSPGDYTIRVAAKAMPQLLS
GYAVLSYKKSLLNIKLGGVTSAPPAKHPFIDYGGNVISGSGGVLFNAMAIQPFPAQAPPSGPADVTIRISLQ

RSDALTWTMNGVPLPTLPEQFTPLLIDPKGISNLDPALYTSFKNGSIVDIILETTVLGKGSHPAHPIHKHNTK
AFFLGTGTGRFPAATVWEAFQRNTTGLNLVNPPLRDDFNTPPTKENTNWAALRFQSVNPMVTIMHCHID
PHLAGGMSFIMMEGLEALQPIPAYYKDFH
